# Sex differences in cancer survival in Estonia: a population-based study

**DOI:** 10.1186/s12885-015-1080-9

**Published:** 2015-02-19

**Authors:** Kaire Innos, Peeter Padrik, Vahur Valvere, Tiiu Aareleid

**Affiliations:** 1 Department of Epidemiology and Biostatistics, National Institute for Health Development, Hiiu 42, 11619 Tallinn, Estonia; 2 Clinic of Haematology and Oncology, Tartu University Hospital, Tartu, Estonia; 3 Clinic of Haematology and Oncology, University of Tartu, Tartu, Estonia; 4 Clinic of Oncology and Haematology, North Estonia Medical Centre, Tallinn, Estonia; 5 Competence Centre for Cancer Research, Tallinn, Estonia

**Keywords:** Relative survival, Cancer registry, Population-based, Gender differences, Data quality

## Abstract

**Background:**

In Estonia, women have much longer life expectancy than men. The aim of this study was to examine sex differences in cancer survival in Estonia and to explore the role of age at diagnosis, stage at diagnosis and tumour subsite.

**Methods:**

Using data from the population-based Estonian Cancer Registry, we examined the relative survival of adult patients diagnosed with nine common cancers in Estonia in 1995–2006 and followed up through 2011. Excess hazard ratios (EHR) of death associated with female gender adjusted for age, stage at diagnosis and tumour subsite were estimated.

**Results:**

A total of 20 828 male and 13 166 female cases were analysed. The main data quality indicators were similar between men and women. Women had more cases with unknown extent of disease at diagnosis. Overall, the age-adjusted 5-year relative survival ratio was higher among women than men for all studied sites, but the difference was significant for cancers of mouth and pharynx (22% units), lung (5% units), skin melanoma (17% units) and kidney (8% units). The increase in survival over time was larger for women than men for cancers of mouth and pharynx, colon, rectum, kidney and skin melanoma. In multivariate analysis, women had a significantly lower EHR of death within five years after diagnosis for five of the nine cancers studied (cancers of mouth and pharynx, stomach, lung, skin melanoma and kidney). Adjustment for stage and subsite explained some, but not all of the women’s advantage.

**Conclusions:**

We found a significant female survival advantage in Estonia for cancers of mouth and pharynx, stomach, lung, kidney and skin melanoma. The differences in favour of women tended to increase over time as from the 1990s to the 2000s, survival improved more among women than among men. A large part of the women’s advantage is likely attributable to biological factors, but other factors, such as co-morbidities, treatment compliance or health behaviour, are also probable contributors to gender survival disparities in Estonia and merit further investigation. Our findings have implications for public health, early detection and cancer care in Estonia.

## Background

Women have a longer life expectancy than men; in 2012, the difference between the life expectancy at birth of women and men in Europe was 5.6 years (83.1 vs. 77.5 years, respectively) [1]. In Estonia, health disparities between sexes are even larger, as the life expectancy at birth is 81 years for women, but only 73 years for men [2]. Among all causes of death, cancer ranks second after circulatory diseases [3]. Globally, cancer mortality among men is much higher compared to women [4]. Mostly, this difference mirrors sex differences in cancer incidence [5], but many studies have also observed sex disparities in cancer survival [6,7]. As hypothesised elsewhere, a considerable part of gender differences in cancer survival likely arises from inequalities in tumour biology, including host defence mechanisms, while the factors such as health behaviour, awareness of symptoms, timeliness of diagnosis and co-morbidity may also be important contributors to gender disparities [8].

The aim of this study was to analyse gender differences in cancer survival in Estonia and to explore the role of age at diagnosis, stage at diagnosis and tumour subsite using data from the population-based Estonian Cancer Registry (ECR). Cancer is a reportable disease in Estonia by legislation. The registry covers the whole country (population 1.34 million in 2009) and has population-based cancer data since 1968 [9]. Estonian cancer data have been included in the Cancer Incidence in Five Continents since Volume VI (data from 1983).

## Methods

The ECR provided individual data with no personal identifiers on all adult (15 years and older) cases of nine most common malignancies occurring in both genders, diagnosed in Estonia in 1995–2006, regardless of cancer sequence: mouth and pharynx (ICO-O-3 topography codes C00–C14); stomach (C16), colon (C18), rectum, rectosigmoid junction, anus (C19–C21), pancreas (C25), lung, trachea (C33–C34), melanoma of skin (C44), kidney, renal pelvis (C64–C65), bladder, ureter, other urinary organs (C66–C68). The following quality indicators were calculated: percentage microscopically verified (%MV), percentage death certificate only (%DCO), percentage autopsy and percentage unknown extent of disease. Cases diagnosed at autopsy and DCO cases were excluded from survival analysis. The patients were followed up for vital status until December 31, 2011 using linkage with the Population Registry. This routine linkage is based on unique personal identification numbers [10]. In case of death or emigration, the respective date was ascertained. Relative survival was calculated as the ratio of the observed survival of cancer patients and the expected survival of the underlying general population. The latter was calculated according to the Ederer II method using national life tables for Estonian population stratified by age, gender and calendar year. The patients were grouped into five age categories: 15–44 years, 45–54 years, 55–64 years, 65–74 years, ≥75 years. The two youngest age groups were collapsed for the modelling of excess mortality except for skin melanoma. Subsites according to ICD-O-3 were considered for five cancers (mouth and pharynx, stomach, colon, rectum, skin melanoma), for which subsite may be a prognostic factor based on a priori knowledge. Extent of disease was grouped into four categories based on the information reported to the cancer registry: 1) localised; 2) local/regional spread (regional lymph nodes or adjacent tissues); 3) distant (distant metastases or unspecified advanced process); 4) unknown extent. For age adjustment, we used the International Cancer Survival Standards (ICSS) [11]. Age-adjusted relative survival ratios (RSR) are presented for all patients and separately for patients diagnosed in 1995–2000 and 2001–2006.

The significance of the difference between proportions was tested using a chi-square test. We estimated excess hazard ratios (EHR) of death associated with female gender within five years after diagnosis in the framework of generalized linear models using a Poisson assumption for the number of observed deaths [12]. Cases with unknown extent of disease were excluded from these analyses. All models included year of follow-up and period of diagnosis. The EHRs are presented with 95% confidence intervals (95% CI). Interactions between year of follow-up and stage or subsite were checked with likelihood ratio test.

Stata 12 software was used for all analyses. The study protocol was approved by the Tallinn Medical Research Ethics Committee (decision no. 284).

## Results

A total of 20 828 male and 13 166 female cases of nine cancers sites were analysed. The differences between men and women in the main data quality indicators were generally small (Table 1). In both male and female patients, %MV varied across cancer sites with the lowest result for pancreas and lung. For the same sites, %DCO exceeded 3% in both sexes. Skin melanoma had the highest %MV and the lowest %DCO. Among cased diagnosed alive, the proportion of cases with unknown extent of disease was higher in women for all sites except mouth and pharynx, and exceeded 5% for cancers of pancreas, lung, stomach and bladder (only pancreas in men). Female patients were older than male patients: median age at diagnosis as well as the respective 75th percentile was higher among women for all sites. Age distribution by cancer site is shown in Table 2.

**Table 1:** **Nine common cancers in Estonia, 1995–2006**

	Men						Women					
Cancer site	No. of cases	%MV	%DCO	%Autopsy	%Unknown extent*	Age, median (IQR)*	No. of cases	%MV	%DCO	%Autopsy	%Unknown extent*	Age, median (IQR)*
Mouth, pharynx	1410	94.5	0.8	0.2	1.6	61 (54–68)	457	94.8	0.7	0.2	1.6	68 (57–76)
Stomach	3003	90.0	1.7	1.8	3.6	67 (58–73)	2505	88.5	2.4	1.3	5.3	71 (61–77)
Colon	2029	91.4	1.4	1.3	2.7	69 (62–75)	2789	88.5	1.2	1.6	3.9	72 (64–78)
Rectum	1627	93.6	1.0	0.5	2.6	68 (61–74)	1622	90.2	1.5	0.9	3.7	71 (63–78)
Pancreas	1235	53.7	3.1	4.5	6.1	66 (58–73)	1099	47.3	3.6	2.2	7.5	72 (64–79)
Lung	7442	73.6	3.5	3.2	4.3	66 (60–72)	1771	66.9	3.4	3.6	5.8	70 (62–76)
Skin melanoma	539	98.5	0.7	0.2	2.8	62 (51–71)	980	99.1	0.2	0.3	3.0	64 (49–73)
Kidney	1742	81.2	1.8	4.7	2.5	64 (56–71)	1255	80.1	1.0	3.2	4.0	68 (60–75)
Bladder	1801	94.0	1.2	1.0	4.0	69 (63–76)	688	90.0	2.8	1.5	5.5	74 (67–80)

*Abbreviations*: *%MV* percentage microscopically verified, *%DCO* percentage death certificate only, *IQR* interquartile range.

*Among cases diagnosed alive and eligible for survival analysis.

**Table 2:** **Age distribution among cancer cases eligible for survival analysis, Estonia, 1995–2006**

		No. of cases (%)		
Cancer site	Age at diagnosis (years)	Men	Women	p-value*
Mouth, pharynx	<45	76 (5)	35 (8)	p < 0.001
	45–54	317 (23)	60 (13)	
	55–64	503 (36)	92 (20)	
	65–74	379 (27)	128 (28)	
	≥75	120 (9)	136 (30)	
Stomach	<45	140 (5)	126 (5)	p < 0.001
	45–54	377 (13)	248 (10)	
	55–64	756 (26)	440 (18)	
	65–74	1067 (37)	801 (33)	
	≥75	557 (19)	797 (33)	
Colon	<45	54 (3)	73 (3)	p < 0.001
	45–54	159 (8)	183 (7)	
	55–64	456 (23)	498 (18)	
	65–74	809 (41)	970 (36)	
	≥75	496 (25)	988 (36)	
Rectum	<45	44 (3)	56 (4)	p < 0.001
	45–54	134 (8)	131 (8)	
	55–64	428 (27)	307 (19)	
	65–74	641 (40)	531 (34)	
	≥75	356 (22)	227 (35)	
Pancreas	<45	56 (5)	20 (2)	p < 0.001
	45–54	18 (13)	76 (7)	
	55–64	318 (28)	184 (18)	
	65–74	414 (36)	350 (34)	
	≥75	206 (18)	405 (39)	
Lung	<45	132 (2)	43 (3)	p < 0.001
	45–54	785 (11)	159 (10)	
	55–64	2131 (31)	334 (20)	
	65–74	2859 (41)	610 (37)	
	≥75	1041 (15)	501 (30)	
Skin melanoma	<45	98 (18)	188 (19)	p = 0.076
	45–54	75 (14)	147 (15)	
	55–64	130 (24)	199 (20)	
	65–74	147 (28)	239 (25)	
	≥75	83 (16)	199 (20)	
Kidney	<45	86 (5)	38 (3)	p < 0.001
	45–54	286 (18)	130 (11)	
	55–64	472 (29)	302 (25)	
	65–74	544 (34)	433 (36)	
	≥75	229 (14)	290 (24)	
Bladder	<45	45 (3)	18 (3)	p < 0.001
	45–54	137 (8)	33 (5)	
	55–64	388 (22)	86 (13)	
	65–74	702 (40)	212 (32)	
	≥75	488 (28)	310 (47)	

*Chi-squared test.

Subsite distribution varied between sexes for all five cancers (Table 3). Stage distribution differed significantly for cancers of mouth and pharynx, stomach, lung, kidney and skin melanoma (Table 4).

**Table 3:** **Subsite distribution among cancer cases eligible for survival analysis, Estonia, 1995–2006**

			No of cases (%)		
Cancer site	Subsite	ICD-O-3	Men	Women	p-value*
Mouth, pharynx	All	C00–14	1395 (100)	451 (100)	p < 0.001
	Lip	C00	158 (11)	77 (17)	
	Tongue and oral cavity	C01–06	591 (42)	203 (45)	
	Salivary glands	C07–08	95 (7)	96 (21)	
	Pharynx	C09–13	529 (38)	70 (16)	
	Other ill-defined sites	C14	22 (2)	5 (1)	
Stomach	All	C16	2897 (100)	2412 (100)	p < 0.001
	Cardia	C16.0	249 (9)	136 (6)	
	Non-cardia	C16.1–16.4	1553 (54)	1308 (54)	
	Other	C16.5–16.8	238 (8)	223 (9)	
	Unspecified	C16.9	857 (29)	745 (31)	
Colon	All	C18	1974 (100)	2712 (100)	p < 0.001
	Right	C18.0–18.3	630 (32)	1055 (39)	
	Transverse	C18.4	181 (9)	308 (11)	
	Left	C18.5–18.7	1089 (55)	1263 (47)	
	Overlapping	C18.8	38 (2)	29 (1)	
	Unspecified	C18.9	36 (2)	57 (2)	
Rectum	All	C19–21	1603 (100)	1582 (100)	p < 0.001
	Rectosigmoid junction	C19	234 (14)	253 (16)	
	Rectum	C20	1326 (83)	1229 (78)	
	Anus, anal canal	C21	43 (3)	100 (6)	
Skin melanoma	All	C44	533 (100)	972 (100)	p < 0.001
	Head and neck	C44.0–44.4	74 (14)	133 (14)	
	Trunk	C44.5	280 (52)	280 (29)	
	Upper limbs	C44.6	54 (10)	147 (15)	
	Lower limbs	C44.7	78 (15)	352 (36)	
	Overlapping, unspecified	C44.8–44.9	47 (9)	60 (6)	

*Chi-squared test.

**Table 4:** **Stage distribution among cancer cases eligible for survival analysis, Estonia, 1995–2006**

		No. of cases (%)		
Cancer site	Extent	Men	Women	p-value*
Mouth, pharynx	All	1395 (100)	451 (100)	p < 0.001
	Localized	564 (40)	256 (57)	
	Local/regional spread	752 (54)	169 (37)	
	Distant	57 (4)	19 (4)	
	Unknown	22 (2)	7 (2)	
Stomach	All	2897 (100)	2412 (100)	p < 0.001
	Localized	650 (22)	616 (26)	
	Local/regional spread	739 (26)	579 (24)	
	Distant	1404 (48)	1090 (45)	
	Unknown	104 (4)	127 (5)	
Colon	All	1974 (100)	2712 (100)	p = 0.048
	Localized	787 (40)	1039 (38)	
	Local/regional spread	509 (26)	748 (28)	
	Distant	625 (32)	819 (30)	
	Unknown	53 (3)	106 (4)	
Rectum	All	1603 (100)	1582 (100)	p = 0.099
	Localized	681 (42)	637 (40)	
	Local/regional spread	449 (28)	479 (30)	
	Distant	432 (27)	407 (26)	
	Unknown	41 (3)	59 (4)	
Pancreas	All	1142 (100)	1035 (100)	p = 0.114
	Localized	117 (10)	132 (13)	
	Local/regional spread	271 (24)	245 (24)	
	Distant	684 (60)	580 (56)	
	Unknown	70 (6)	78 (7)	
Lung	All	6948 (100)	1647 (100)	p = 0.003
	Localized	1127 (16)	285 (17)	
	Local/regional spread	2676 (39)	567 (34)	
	Distant	2846 (41)	699 (42)	
	Unknown	299 (4)	96 (6)	
Skin melanoma	All	533 (100)	972 (100)	p < 0.001
	Localized	360 (68)	761 (78)	
	Local/regional spread	98 (18)	108 (11)	
	Distant	60 (11)	74 (8)	
	Unknown	15 (3)	29 (3)	
Kidney	All	1617 (100)	1193 (100)	p < 0.001
	Localized	876 (54)	728 (61)	
	Local/regional spread	228 (14)	145 (12)	
	Distant	473 (29)	272 (23)	
	Unknown	40 (3)	48 (4)	
Bladder	All	1760 (100)	659 (100)	p = 0.299
	Localized	1325 (75)	486 (74)	
	Local/regional spread	213 (12)	87 (13)	
	Distant	152 (9)	50 (8)	
	Unknown	70 (4)	36 (5)	

*Chi-squared test.

Women had a significant survival advantage for cancers of mouth and pharynx, lung, skin melanoma and kidney (Table 5). For these sites, women also experienced superior survival in all age groups, although the advantage was generally larger among younger patients and statistically significant differences were seen only in some age categories. For bladder cancer, women had worse prognosis (non-significant) only among patients younger than 55 years.

**Table 5:** **Five-year relative survival ratios (RSR) in Estonia, women (W) and men (M), 1995–2006**

	All ages (age-adjusted)			15–44			45–54			55–64			65–74			≥75		
Cancer site	RSR (W) %	RSR (M) %	RSR(W) minus RSR(M)	RSR (W) %	RSR (M) %	RSR(W) minus RSR(M)	RSR (W) %	RSR (M) %	RSR(W) minus RSR(M)	RSR (W) %	RSR (M) %	RSR(W) minus RSR(M)	RSR (W) %	RSR (M) %	RSR(W) minus RSR(M)	RSR (W) %	RSR (M) %	RSR(W) minus RSR(M)
Mouth, pharynx	52	30	22*	62	31	31*	55	26	29*	54	22	32*	48	37	11	51	30	21
Stomach	23	20	3	26	31	−5	29	22	7	28	24	4	23	18	5	16	16	0
Colon	51	50	1	58	60	−2	52	53	−1	52	47	5	52	52	0	46	46	0
Rectum	46	42	4	42	42	0	53	41	12	53	50	3	49	48	1	34	31	3
Pancreas	6	5	1	15	17	−2	12	5	7	5	6	−1	5	2	3	3	5	−2
Lung	14	9	5*	28	15	13	23	10	13*	15	10	5	9	8	1	11	6	5
Skin melanoma	73	56	17*	82	66	16*	75	60	15	69	51	18*	71	60	11	64	36	28
Kidney	62	54	8*	82	64	18	77	56	21*	69	58	11*	56	56	0	51	45	6
Bladder	65	63	2	78	88	−10	72	80	−8	73	66	7	67	60	7	50	50	0

*Significant difference at the 0.05 level.

During the first period (1995–2000), the age-adjusted five-year RSR was higher among women for all sites except colon (Table 6); the women’s advantage was statistically significant for mouth and pharyngeal cancer (20% units) and lung cancer (6% units), while it was borderline significant for skin melanoma (14% units). During the second period (2001–2006), the women’s advantage increased to 24% units for cancer of mouth and pharynx and 19% units for skin melanoma (both statistically significant). As kidney cancer survival increased only among women, the gender difference reached a significant difference of 13% units during the second period. For lung cancer, the 6% unit significant difference persisted throughout the study period. The increase in survival over time was much larger for women than men for both colon and rectal cancers.

**Table 6:** **Age-adjusted five-year relative survival ratios (RSR) in Estonia, 1995–2006**

	Men		Women			
	5-year RSR (95% CI)		5-year RSR (95% CI)		Difference, 1995–2000*	Difference, 2001–2006*
Cancer site	1995–2000	2001–2006	1995–2000	2001–2006		
Mouth, pharynx	28 (22–34)	32 (26–37)	48 (40–56)	56 (48–63)	20**	24**
Stomach	20 (18–23)	20 (18–23)	23 (20–25)	24 (21–27)	3	4
Colon	52 (47–57)	47 (43–51)	49 (46–52)	53 (49–56)	−3	6
Rectum	38 (33–42)	46 (42–50)	39 (35–43)	52 (48–56)	1	6
Pancreas	5 (3–8)	4 (3–7)	7 (4–10)	4 (3–7)	2	0
Lung	7 (6–9)	10 (9–12)	13 (10–16)	16 (13–19)	6**	6**
Skin melanoma	58 (50–66)	56 (49–62)	72 (66–76)	75 (70–79)	14	19**
Kidney	54 (48–60)	54 (49–59)	58 (52–62)	67 (62–71)	4	13**
Bladder	62 (57–67)	63 (59–67)	64 (57–70)	66 (59–72)	2	3

*Five-year RSR among women minus respective RSR among men.

**Significant difference at the 0.05 level.

Women had a significantly lower EHR of death within five years of diagnosis for five of the nine cancers studied (cancers of mouth and pharynx, stomach, lung, skin melanoma and kidney) (Table 7). In age-adjusted analyses, women had almost 50% lower EHR of dying compared to men for cancers of mouth and pharynx and skin melanoma. Adjustment for stage and subsite explained some of the women’s advantage, but a significant portion of this advantage remained unexplained. For stomach cancer, women had 10% lower EHR of death and the inclusion of stage and subsite into the model did not change the estimates. For cancers of lung and kidney, women still had around 20% lower EHR of dying compared to men after adjustment for stage. We observed no survival differences between men and women in any of the models for cancers of colon, rectum, pancreas and bladder.

**Table 7:** **Excess hazard ratio (EHR) of death within five years after diagnosis for women compared to men, Estonia, 1995–2006**

Cancer site	EHR (95% CI)*	EHR adjusted for age (95% CI)	EHR adjusted for age and extent (95% CI)	EHR adjusted for age, extent and subsite** (95% CI)
Mouth, pharynx	0.54 (0.46–0.63)	0.54 (0.46–0.64)	0.60 (0.51–0.71)	0.66 (0.56–0.78)
Stomach	0.92 (0.86–0.98)	0.88 (0.82–0.94)	0.90 (0.84–0.96)	0.91 (0.85–0.97)
Colon	1.05 (0.96–1.15)	1.01 (0.92–1.11)	1.10 (1.00–1.21)	1.09 (0.99–1.19)
Rectum	0.98 (0.88–1.10)	0.92 (0.83–1.03)	1.00 (0.90–1.11)	0.99 (0.89–1.10)
Pancreas	1.00 (0.91–1.09)	0.94 (0.85–1.03)	0.97 (0.88–1.06)	
Lung	0.90 (0.85–0.96)	0.87 (0.82–0.93)	0.81 (0.76–0.86)	
Skin melanoma	0.53 (0.43–0.66)	0.52 (0.43–0.64)	0.68 (0.55–0.84)	0.72 (0.58–0.90)
Kidney	0.76 (0.67–0.87)	0.72 (0.63–0.83)	0.81 (0.70–0.93)	
Bladder	1.03 (0.86–1.24)	0.92 (0.77–1.11)	0.96 (0.80–1.15)	

*All models include year of follow-up and period of diagnosis.

**Mouth, pharynx: lip (ICD–O–3 C00), oral cavity and tongue (C01-06), salivary glands (C07–08), pharynx and other (C09–14); Stomach: cardia (C16.0), non-cardia (C16.1–16.4), other (C16.5–16.9); Colon: right (C18.0–18.3), transverse (C18.4), left (C18.5–18.7), other (C18.8–18.9); Rectum: rectosigmoid junction (C19), rectum (C20), anus and anal canal (C21); Skin melanoma: head and neck (C44.0–44.4), trunk (C44.5), upper limbs (C44.6), lower limbs (C44.7), other (C44.8–44.9).

We found evidence of interaction between year of follow-up and extent of disease (stomach, rectum, pancreas, lung melanoma, kidney and bladder) or year of follow-up and age (stomach, colon, rectum, lung, bladder); however, including the interaction term in the model did not change the risk estimates for gender, and therefore, these results are not shown.

## Discussion

In this study based on data from the population-based cancer registry of Estonia, we found that women had a significant survival advantage for five of the nine common solid tumours included in the study. The direction and magnitude of the effect found in our study is consistent with the findings of the analysis of EUROCARE-4 data for cancers of mouth and pharynx, stomach, lung, and skin melanoma [6]. For kidney cancer, the women’s advantage observed in our study was much larger than in the European pool. Unlike the EUROCARE-4 analysis, we did not find female survival advantage for cancers of colon, rectum and pancreas. For bladder cancer, we found no gender differences in survival whilst a significant female *disadvantage* was seen in the EUROCARE-4 analysis.

Age is a major determinant of survival [13]. We found that for most cancers, the gender difference in survival varied across age categories; for many sites, the women’s advantage was more marked in younger age groups. It has been suggested that in this context, age is a proxy for biological factors, particularly profound hormonal changes that occur in women around the age of menopause [6]. On the other hand, in younger and middle-aged men free testosterone is hypothesised to drive cancer aggressiveness [14].

The main strength of the study was the use of good-quality population-based data, collected uniformly over the study period. The quality of the ECR has remained relatively stable from 1995 to 2008 with low %DCO and percentage primary site uncertain [15]. In this study, we found that the main quality indicators did not vary notably by sex for any cancer site. Nevertheless, female patients were generally older than male patients, with about 2-fold higher proportion of the age group 75 years and older for some cancers (mouth and pharynx, pancreas and bladder). This was probably the main reason for the somewhat higher proportion of cases with unknown extent of disease observed among women compared with men. As an additional strength of the study, we were able to account for two major determinants of survival – cancer subsite and extent of disease.

The main limitation of the study was the inability to examine the role of determinants of survival other that age, stage and subsite, such as co-morbidities, health behaviour or factors associated with tumour biology. The extent of disease at diagnosis as reported to the ECR is not as precise as TNM stage and some misclassification is possible. Misclassification of cancer subsite needs to be considered as well. However, we do not expect any of the misclassifications to be associated with gender. Relative survival compensates for general background mortality; nevertheless, for patients with tobacco-related cancers, relative survival may be underestimated because their mortality from other diseases such as cardiovascular diseases is higher than in the general population. The prevalence of smoking in men is higher than in women: according to the biannual Estonian Health Behaviour study, the prevalence of daily smokers in men age 15–64 years decreased from 45% in 1996 to 36% in 2012 (from 32% to 26% in women) [16]. Thus, some of the male disadvantage might be due to increased background mortality from smoking-related co-morbidities.

Among oropharyngeal cancers, tongue and oral cavity was the most common subsite for both genders, accounting for 2/5 of the cases. The second most frequent subsite was pharynx among men (ICD-O-3 C09–C13), but salivary gland (ICD-O-3 C07–C08) among women. Based on the EUROCARE study, the summary age-adjusted 5-year RSR was significantly higher for cancers of salivary glands than pharynx (especially hypopharynx) [13]. Pharyngeal subsites were associated with considerably higher excess risk of dying compared with subsites within oral cavity in an analysis of head and neck cancers in Europe [17]. Thus, different proportions of these subsites can contribute by itself to survival advantage among women. Our results showed that the women’s survival advantage was rather constant over the age-span and only slightly decreased after the age of 65 years. In women, the improvement of 5-year RSR from 1995–2000 to 2001–2006 was more rapid compared to men, and as a result, the gender difference increased. Women also had more favourable stage distribution (higher proportion of localised disease). In multivariate analysis, however, considerable women’s survival advantage persisted even after adjustment for age, stage and subsite. Socioeconomic status has been shown to influence survival [18]. Co-morbidities, treatment compliance and health behaviour after diagnosis may also play a role, taking into consideration the major risk factors for these cancers, particularly smoking and alcohol consumption. Head and neck cancers associated with human papillomavirus (HPV) have better survival than HPV-negative tumours, which are mainly alcohol- or tobacco-related [19], but no data are available regarding the HPV prevalence in oropharyngeal tumours in Estonia.

For stomach cancer, we observed a 3–4% unit female survival advantage, similar to that found in Germany and US [20]. Women had 10% lower EHR of dying, which was not influenced by stage or subsite distribution. The analysis of EUROCARE-4 data demonstrated a similar female survival advantage [6]. In the Austrian study, women still had a survival advantage in an age- and stage-adjusted model [21], while in Korea, women’s higher risk of death disappeared after adjusting for age and stage [22]. Several underlying mechanisms have been proposed for the protective effect of oestrogens against gastric cancer [23]. The presence of oestrogen receptors could protect against invasiveness of stomach cancer [24], but their prognostic role may be limited [25]. Body mass index (BMI) could be important in surgical outcomes, especially in men: male patients with high BMI were shown to have longer duration of operation and larger extent of bleeding compared to patients with lower BMI; no such difference was seen among female patients [26].

In contrast to some previous studies [5,6,27], we did not find female survival advantage for colon and rectum cancers. The reasons for this are not clear. There was a slight tendency towards female advantage during the most recent period, which warrants further analysis. The stage distributions did not differ between men and women in our study. For colon cancer, there was left predominance of colon cancer among men and right-transverse predominance among women; this pattern has been observed previously [27]. Several previous studies found that the women’s advantage was age-related. For example, in the EUROCARE analysis, the women’s advantage was much larger in the age group 15–54 years [6] and in a SEER-based study, women had better survival than men until the age of 64 years [28]. In Germany, female advantage was most notable in younger-middle ages and the advantage varied by stage of CRC (larger for localised cancers) [27]. We did not find any age-related patterns in survival differences, possibly due to small numbers in younger age groups.

Women had 6% units higher 5-year RSR for lung cancer in both periods, but the largest difference (13% units) was seen amongst patients younger than 55 years of age. In the EUROCARE-4-based study, a significant difference between the age-specific relative survival of men and women was seen only among patients younger than 65 years [6]. After adjusting for age and stage, women in our study had nearly 20% lower EHR of dying than men. This estimate is very close to that found in an Austrian study after similar adjustment [21]. The proportion of small-cell lung cancer was 16% in both men and women; women had a higher proportion of adenocarcinomas and men had more squamous cell carcinomas, but the inclusion of this variable into the model had no considerable effect on the EHR of dying (data not shown). In a Dutch study, men with lung cancer had higher prevalence of co-morbid conditions than women; it was also shown that patients with co-morbidities (particularly the elderly) were treated less aggressively [29]. On the other hand, a SEER-based study of treated and untreated elderly patients suggested a different natural history and different tumour biology of lung cancer in men and women [30]. In addition, genetic and hormonal have been proposed as a potential explanation for the survival benefit experienced by women [31].

The absolute difference between the 5-year RSR of male and female melanoma patients increased from 14% units to 19% units from 1995–2000 to 2001–2006 as survival did not improve in men. The 5-year RSR for women in Estonia in 2001–2006 was 12% units lower than the European mean for 2000–2007 reported in EUROCARE-5 (75% vs. 87%), but the respective difference among men was as much as 23% units (56% vs. 79%) [13]. We found female advantage in all age groups in our study, with a particularly large difference among patients age 75 years and over. The predominant subsite was trunk among men, but lower limbs among women. Women were more likely to have localised melanoma. In age-adjusted analysis, female patients had almost 50% lower EHR of death and this is consistent with the results of the analysis on the European pool [6]. Although men were more likely to have melanomas with unfavourable characteristics, such as location on the trunk and more advanced stage at diagnosis, these factors did not fully explain the women’s survival advantage. In a large Dutch population-based study, men were nearly twice as likely to die from their melanoma compared with females after adjusting for a number of patient and tumour characteristics such as age, region, Breslow thickness, histology, body site as well as nodal and metastatic status [32]. In a Finnish study, male patients had worse survival than females in all site groups [33]. The persistent female survival advantage among cases with pathology confirmed advanced disease suggested different immunological response to melanoma [32]. At the same time, the unfavourable effect of lower socioeconomic status on melanoma survival was also more pronounced among men than among women [34].

Female kidney cancer patients had around 20% lower EHR of dying than males in our study. This difference is larger than seen in the EUROCARE-4 analysis [6]. The 5-year RSR for women in Estonia in 2001–2006 was 5% units higher than the respective European mean in EUROCARE-5 analysis (67% vs. 62%), but for men the difference was 6% in favour of the European mean (54% vs. 60%) [13]. Some previous studies have shown worse survival for women [22] or no difference [21] while many studies have reported women’s survival advantage [35]. Kidney cancer survival improved over the study period only among women in Estonia. Women showed more favourable stage distribution which may be related to the increasing use of modern abdominal imaging techniques. Greater use of diagnostic imaging may result in the diagnosis of more incidental renal cell carcinomas among females than males and these tumours have better prognosis compared with symptomatic tumours [35]. The comparison of age-specific RSRs revealed that women’s significant survival advantage was limited to patients under the age of 65 years.

Previous population-based studies have found poorer outcomes for bladder cancer in women compared to men [5,6,21,22]. We found non-significantly higher RSRs for men among patients younger than 55 years, but overall analysis did not reveal any differences. The male advantage has been associated with different management and referral practices [36,37]. In Estonia, women visit doctors much more often than men; the proportion of men who had visited a specialist physician during the past year has been consistently about 18% units lower than that of women (33% vs. 51% in 2002 and 41% vs. 59% in 2012) [16].

## Conclusions

The age-adjusted 5-year RSR was higher among women than men for all nine cancer sites included in the study. A statistically significant female advantage in the 5-year RSR was seen for cancers of mouth and pharynx, lung, kidney and skin melanoma. The differences in favour of women tended to increase over time, as survival improved more among women than among men over the study period. For several sites, the survival gap between the most recent estimates for Estonia and the rest of Europe was much larger for men than for women.

Age was a significant contributor to gender survival differences, but its role was not uniform across the cancer sites studied. For lung and kidney cancer, the women’s advantage was largest before age 55 years, suggesting the role of hormonal factors; for mouth and pharyngeal cancer and melanoma, the advantage was very large until age 65 and increased again in patients age 75 years and older, suggesting the combined effect of biological and social factors as well as co-morbidities. For five sites (mouth and pharynx, stomach, lung, skin melanoma and kidney), women had a significantly lower EHR of dying in multivariate analysis that was not explained by age, stage or subsite.

A large part of the women’s advantage is likely attributable to biological factors, including hormonal status or different molecular subtypes. The magnitude of the women’s advantage observed in this study suggests that the other factors, such as co-morbidities, treatment compliance and/or health behaviour (including degree of change in health behaviour after diagnosis) could be contributors to gender disparities in Estonia and merit further investigation using high-resolution approach. As some of these factors are modifiable, our findings have implications for public health, early detection and cancer care strategies in Estonia.
